# Lymphangiogenesis and Angiogenesis in Abdominal Aortic Aneurysm

**DOI:** 10.1371/journal.pone.0089830

**Published:** 2014-03-20

**Authors:** Masaki Sano, Takeshi Sasaki, Satoshi Hirakawa, Junichi Sakabe, Mikako Ogawa, Satoshi Baba, Nobuhiro Zaima, Hiroki Tanaka, Kazunori Inuzuka, Naoto Yamamoto, Mitsutoshi Setou, Kohji Sato, Hiroyuki Konno, Naoki Unno

**Affiliations:** 1 Division of Vascular Surgery, Applied Medical Photonics Laboratory, Medical Photonics Research Center, Hamamatsu City, Shizuoka, Japan; 2 Department of Anatomy and Neuroscience, Applied Medical Photonics Laboratory, Medical Photonics Research Center, Hamamatsu City, Shizuoka, Japan; 3 Department of Dermatology, Applied Medical Photonics Laboratory, Medical Photonics Research Center, Hamamatsu City, Shizuoka, Japan; 4 Department of Molecular Imaging, Applied Medical Photonics Laboratory, Medical Photonics Research Center, Hamamatsu City, Shizuoka, Japan; 5 Department of Diagnostic Pathology, Hamamatsu University School of Medicine, Hamamatsu, Shizuoka, Japan; 6 Department of Cell Biology and Anatomy, Hamamatsu University School of Medicine, Hamamatsu, Shizuoka, Japan; 7 Second Department of Surgery, Hamamatsu University School of Medicine, Hamamatsu, Shizuoka, Japan; 8 Department of Applied Biological Chemistry, Kinki University, Osaka, Japan; University of Louisville, United States of America

## Abstract

The pathogenesis of abdominal aortic aneurysm (AAA) is characterized to be inflammation-associated degeneration of vascular wall. Neovascularization is regularly found in human AAA and considered to play critical roles in the development and rupture of AAA. However, little is known about lymphangiogenesis in AAA. The purpose of this study was to demonstrate both angiogenesis and lymphangiogenesis in AAA. Abdominal aortic tissue was harvested either from autopsy (control group) and during open-repair surgery for AAA (AAA group). Adventitial lymphatic vasa vasorum was observed in both groups, but seemed to be no significant morphological changes in AAA. Immunohistochemical studies identified infiltration of lymphatic vessel endothelial hyaluronan receptor (LYVE) −1, vascular endothelial growth factor (VEGF)-C, and matrix metalloproteinase (MMP)-9-positive macrophages and podoplanin and Prox-1-positive microvessels in the intima/media in AAA wall, where hypoxia-inducible factors (HIF)-1α was expressed. VEGF-C and MMP-9 were not expressed in macrophages infiltrating in the adventitia. Intraoperative indocyanine green fluorescence lymphography revealed lymph stasis in intima/medial in AAA. Fluorescence microscopy of the collected samples also confirmed the accumulation of lymph in the intima/media but not in adventitia. These results demonstrate that infiltration of macrophages in intima/media is associated with lymphangiogenesis and angiogenesis in AAA. Lymph-drainage appeared to be insufficient in the AAA wall.

## Introduction

Abdominal aortic aneurysm (AAA) is a common disease among elderly people. AAA affects 5–10% of men over 65 years and is the tenth leading cause of death in men over the age of 55 years in the United States [1]. When surgical treatment is inapplicable, AAA progress to rupture with a high mortality (30–50%). An effective nonsurgical therapy is currently not available, because the precise mechanisms of AAA pathogenesis is yet to be identified. The degeneration of vascular wall has been considered as one of the main causes of AAA onset and rupture. Previous studies have demonstrated infiltration of inflammatory cells, such as macrophages, T cells, neutrophils and dendric cells, into the aortic wall [2], [3]. These inflammatory cells are considered to contribute to the pathogenesis of AAA through the secretion of inflammatory mediators, including cytokines, chemokines, and MMPs [4]. In AAA, an intraluminal thrombus prevents luminal perfusion of oxygen, allowing only the adventitial vasa vasorum (VV) to deliver oxygen and nutroents to the aortic wall. We have recently demonstrated the arteriosclerotic degeneration of VV and tissue ischemia in AAA wall [5]. These inflammatory and hypoxic environment are potential stimuli for angiogenesis as previously reported [6]. Angiogenesis is thought to contribute to destructive processes within the AAA wall and plays a key role in aortic aneurysm development and rupture [7]. In various chronic inflammatory situations such as asthma, atopic dermatitis, rheumatoid arthritis, and inflammatory bowel diseases, both angiogenesis and lymphangiogenesis occur simultaneously to glow neovessels to remodel vascular structure [8]–[10]. However, little is known about lymphangiogenesis in AAA. Here, we investigated the presence of lymphanagiogenesis and major drivers of angiogenesis and lymphangiogenesis such as VEGF-A and VEGF-C in AAA wall using AAA samples which were obtained during open repair of AAA surgery.

## Materials and Methods

### Sample collection

All procedures used in this study and the use of samples obtained at autopsy were approved by the Ethics Committee of Clinical Research of the Hamamatsu University School of Medicine. We enrolled 20 consecutive patients who underwent elective open surgery to repair infrarenal AAAs at the Division of Vascular Surgery, Hamamatsu University School of Medicine, between May 2010 and August 2013 and obtained written informed consent for use of the sample from each patient. The aortic tissue was dissected during surgery. Longitudinal tissue strips were resected from the infrarenal aortic neck to the bifurcation.

Twenty aorta obtained at autopsy were used as controls. Written informed consent was also obtained from the donor-family for use of the sample with approval of the Ethics Committee of Clinical Research of the Hamamatsu University School of Medicine. The mid-portion of the abdominal aorta between the renal artery and the bifurcation was resected and collected from routine autopsies between October 2011 and February 2013 in the Department of Pathology, Hamamatsu University Hospital. Autopsy specimens from patients with collagen disease, aortic aneurysm/dissection, or under the age of 18 years were excluded.

Atherosclerotic risk factors (hypertension, hypercholesterolemia, hypertriglyceridemia, diabetes, and smoking) were investigated in AAA patients and autopsy cases. The definition of each item was as follows: hypertension (medication for hypertension or a systolic blood pressure ≧140 mmHg and/or a diastolic blood pressure ≧90 mmHg), hypercholesterolemia (medication for hypercholesterolemia or total cholesterol concentration in the serum of ≧220 mg/dL), hypertriglyceridemia (medication for hypertriglyceridemia or total triglyceride in the serum of ≧150 mg/dL), diabetes (present or past medication for diabetes), smoking (present or past smoking history).

Resected aortic tissues were immersed in 10% neutral buffered formalin for at least 24 h for histological and immunohistochemical staining. These samples were embedded in paraffin; 4 µm sections were cut and mounted onto MAS-coated slides (Matsunami, Osaka, Japan). The aortic tissue was frozen in liquid nitrogen, and fresh frozen tissue was stored at −80°C for quantitative real-time polymerase chain reaction analysis and near-infrared fluorescence microscopy, and immunohistochemical staining.

### Histological and immunohistochemical staining

Paraffin sections were deparaffinized in xylene, rehydrated in solutions of decreasing alcohol concentration, and stained routinely with elastica van Gieson (EVG) staining. Immunohistochemical staining for podoplanin, CD31, vascular endothelial growth factor (VEGF)-A, VEGF-C, vascular endothelial growth factor receptor (VEGFR)-1, VEGFR-2, VEGFR-3, Ki-67, human hypoxia-inducible factor 1 alpha (HIF-1α), macrophages, CD3, CD19, and myeloperoxidase (MPO) were performed. The following primary antibodies were used: mouse monoclonal antibody clone D2-40 (1∶200, DakoCytomation, Glostrup, Denmark) to identify podoplanin in lymphatic endothelial cells, rabbit polyclonal antibody against a synthetic peptide corresponding to the C-terminus of CD31 (1∶100, AnaSpec, CA, USA) to identify vascular endothelial cells, mouse monoclonal antibody against human VEGF-A (1∶50, Abcam, Tokyo, Japan), rabbit polyclonal antibody against human VEGF-C (1∶50, Abcam, Tokyo, Japan), rabbit monoclonal antibody against human VEGFR-1 (1∶100, Epitomics, CA, USA), rabbit polyclonal antibody against human VEGFR-2 (1∶200, Abcam, Tokyo, Japan), rabbit polyclonal antibody against human VEGFR-3 (1∶400, Santa Cruz Biotechnology, CA, USA), rabbit polyclonal antibody against Ki-67 (1∶500, Novus Biologicals, CO, USA), mouse monoclonal antibody against human HIF-1α (1∶100, Novus Biologicals, CO, USA), mouse monoclonal antibody against human macrophages (1∶100, AbD Serotec, Oxford, UK), mouse monoclonal antibody against human CD3 (1∶100, LifeSpan Biosciences, Seatle, WA), mouse monoclonal against human CD19 (1∶50, Santa Cruz Biotechnology, CA, USA), and mouse monoclonal antibody against human myeloperoxidase (MPO) (1∶50, Santa Cruz Biotechnology, CA, USA). The sections were deparaffinized, dehydrated, and boiled in a pressure cooker in 0.01 M citric acid buffer (pH 6.0) for 20 min. The sections were washed with phosphate-buffered saline and incubated with 3% H_2_O_2_ in absolute methanol for 5 min to inhibit any endogenous peroxidase activity. Sections were preincubated with 3% normal goat serum for 20 min to minimize nonspecific binding to the primary antibody, and incubated with the primary antibodies at 4°C overnight in a moist chamber. The sections were washed with phosphate-buffered saline and then incubated with the appropriate secondary antibody for 30 min at room temperature. Staining was visualized with Vector DAB (3,3′-diaminobenzidine, Vector Laboratories, CA, USA), and sections were then counterstained with hematoxylin.

Immunohistochemical staining was also performed on frozen sections. Aortic tissues were embedded in Tissue-Tek OCT compound (Miles Inc., Elkhart, IN, USA), frozen in liquid nitrogen, and stored at −80°C. Frozen sections of 5-µm thickness were prepared using a cryostat (Microm HM560 cryostat, Thermo Scientific, Bremen, Germany), and mounted onto MAS-coated slides (Matsunami, Osaka, Japan). The sections were fixed for 10 min with 4% paraformaldehyde phosphate buffer solution (Wako, Osaka, Japan) and washed with phosphate-buffered saline. The sections were incubated with 3% H_2_O_2_ in absolute methanol for 5 min to inhibit any endogenous peroxidase activity, preincubated with 3% normal goat serum for 20 min to minimize nonspecific binding to the primary antibody, and incubated with the primary antibodies at 4°C overnight in a moist chamber. The sections were washed with phosphate-buffered saline and then incubated with the appropriate secondary antibody for 30 min at room temperature. Staining was visualized with Vector DAB (3,3′-diaminobenzidine, Vector Laboratories, CA, USA), and sections were then counterstained with hematoxylin.

### Double immunofluorescence staining

Colocalization studies were performed with double immunofluorescence staining methods. The following primary antibodies were used: mouse monoclonal antibody against podoplanin (1∶200, DakoCytomation, Glostrup, Denmark), rabbit polyclonal antibody against Prox-1 (1∶2000, Millipore, MA, USA), rabbit polyclonal antibody against the N-terminus of human alpha smooth muscle isoform of actin (1∶25, Thermo Scientific Japan, Tokyo, Japan), mouse monoclonal antibody against human HIF-1α (1∶100, Novus Biologicals, CO, USA), rabbit monoclonal antibody against human CD11b (1∶250, Millipore, MA, USA), mouse monoclonal antibody against human macrophages (1∶100, AbD Serotec, Oxford, UK), rabbit polyclonal antibody against human LYVE-1 (1∶100, Relia Tech, Braunschweig, Germany), rabbit polyclonal antibody against human VEGF-C (1∶50, Abcam, Tokyo, Japan), rabbit polyclonal antibody against human matrix metalloproteinase (MMP)-9 (1∶100, Abnova, Taipei, Taiwan), mouse monoclonal antibody against human CD3 (1∶100, LifeSpan Biosciences, Seattle, WA), mouse monoclonal antibody against human CD19 (1∶50, Santa Cruz Biotechnology, CA, USA), mouse monoclonal antibody against human myeloperoxidase (MPO) (1∶50, Santa Cruz Biotechnology, CA, USA), rabbit polyclonal antibody against transforming growth factor beta-1 (TGF-β1) (1∶50, Abbiotec, CA, USA), rabbit polyclonal antibody against human interleukin-4 (IL-4) (1∶50, Biozol, Munich, Germany), rabbit polyclonal antibody against human interleukin-8 (IL-8) (1∶50, Abnova, Taipei, Taiwan), rabbit polyclonal antibody against human macrophage inflammatory protein-1α (MIP-1α) (1∶50, Spring bioscience, CA, USA), rabbit polyclonal antibody against human interferon-γ (IFN-γ) (1∶50, Santa Cruz Biotechnology, CA, USA), and rabbit polyclonal antibody against human monocyte chemotactic protein-1 (MCP-1) (1∶50, Abnova, Taipei, Taiwan). The sections were deparaffinized, dehydrated, and boiled in a pressure cooker in 0.01 M citric acid buffer (pH 6.0) for 20 min. The sections were washed with phosphate-buffered saline and incubated with 3% H_2_O_2_ in absolute methanol for 5 min to inhibit any endogenous peroxidase activity, preincubated with 3% normal chicken serum for 20 min to minimize nonspecific binding to the primary antibody, and incubated with the primary antibodies at 4°C overnight in a moist chamber. Immunoreactivity was visualized using Alexa Fluor 488-conjugated anti-mouse IgG, Alexa Fluor 594-conjugated anti-mouse IgG, Alexa Fluor 488-conjugated anti-rabbit IgG, and Alexa Fluor 594-conjugated anti-rabbit IgG (Life Technologies, CA, USA). All Alexa Fluor-conjugated secondary antibodies were diluted 200-fold for use. The slides were mounted in glycerol-based Vectashield medium containing the nucleus stain DAPI (Vector Laboratories, CA, USA).

### Morphometric analysis

Immunohistochemical staining for podoplanin was performed to detect lymphatic vessels. Podoplanin was expressed in aortic intima and some podoplanin-positive cells formed microvessels. Lymphatic microvessel (LMV) density and lymphatic microvessel area in the intima were counted per microscopic field [11], [12]. The 10 areas with the highest podoplanin-positive areas within the intima (‘hot spots’) were selected at low magnification (×40) [13]. Lymphatic microvessel density was then determined by counting all immunostained microvessels per ×200 microscopic field using a computerized image analysis system (Lumina Vision version 3.0, Mitani Corp. Tokyo, Japan). The percentage of lymphatic microvessel area, consisting of the podoplanin-positive cells plus the vessel lumen, per ×200 microscopic field (corresponding to an examination area of 0.136 mm^2^) was also measured using the computerized image analysis system [11], [12], [14], [15].

### Near-infrared fluorescence lymphography and microscopy of aneurysmal walls

In AAA patients, near-infrared fluorescence lymphography of the AAA wall was performed intraoperatively. The method was performed as previously described [16].Before open surgical aneurysm repair, 0.3 mL of indocyanine green (ICG; Daiichi Pharmaceutical, Tokyo, Japan; 5 mg/mL saline solution) was subcutaneously injected into the dorsum of each foot. Laparotomy was performed, and the AAA wall was observed with Photodynamic Eye near-infrared camera system (PDE-neo, Hamamatsu Photonics, Hamamatsu, Japan) 2 h after ICG injection. Portions of AAA walls were resected and frozen in liquid nitrogen, and 10 µm frozen sections were cut. The following near-infrared fluorescence microscopy procedure was performed with the use of an upright epifluorescence microscope (Eclipse 80i, Nikon Instruments Inc., NY, USA) with a microscopic near-infrared camera system (Photometrics Evolve 512, Nippon Roper, Tokyo, Japan) to evaluate lymphatic fluid in AAA walls. Fluorescence images were obtained with the ICG fluorescence filter (ICG-B-000, excitation wavelength 769±20.5 nm, emission wavelength 832±18.5 nm, Opto-Line, Tokyo, Japan), antifade reagent (ProLong Gold antifade reagent with DAPI, Life Technologies Japan, Tokyo, Japan), and exposure time 200 µseconds. Pseudocolor images were generated using a computerized image analysis system (Lumina Vision version 3.0, Mitani Corp. Tokyo, Japan). Serial frozen sections of 5 µm thickness were prepared using a cryostat. EVG staining and immunohistochemical staining for podoplanin and macrophages were also performed.

### Quantitative real-time polymerase chain reaction analysis for VEGF and VEGFR gene products

Aortic walls were frozen in liquid nitrogen and stored at −80°C. Fresh frozen aortic tissues were placed into TRIzol reagent (Life Technologies, CA, USA), and homogenized with a mechanical rotor for 1 min. Total mRNA was isolated and purified using the PureLink RNA Mini Kit (Life technologies, CA, USA) according to the protocol for using TRIzol with the PureLink RNA Mini Kit as recommended by the manufacturer. The total RNA concentration of individual sample was determined by spectrophotometer analysis (Nano-drop Technologies, DE, USA) at 260 nm. Reverse transcription was performed using TaqMan Reverse Transcription Reagents (Life Technologies Japan, Tokyo, Japan), and cDNA was prepared from 1000 µg total RNA. Real-time quantitative polymerase chain reaction (PCR) of each sample was carried out with TaqMan Gene Expression Assays and an ABI Prism 7700 Sequence Detection System (Applied Biosystems, CA, USA), based on methods described previously [17]. The Taqman assays used were VEGF-A (Hs00900055_m1), VEGF-C (Hs00153458_m1), c-fos induced growth factor (VEGF-D, Hs01128657_m1), fms-related tyrosine kinase 1 (VEGFR-1, Hs01052961_m1), kinase insert domain receptor (a type III receptor tyrosine kinase, VEGFR-2, Hs00911700_m1), and fms-related tyrosine kinase 4 (VEGF-3, Hs01047677_m1) from Applied Biosystems. Data were analyzed by comparative Ct method [18]. For each sample, the Ct value was divided by that of the housekeeping gene, actin beta (Hs01060665_g1), to generate the standardized Ct value.

### Statistical analysis

Lymphatic microvessel density and the percentage of lymphatic microvessel area in the intima are expressed as means ± SE. Differences between normal aorta and AAA were analyzed by Student's t-test.

Categorical variables were reported as frequencies with percentages, and compared between control and AAA groups using Fisher's exact test to obtain P-values. All continuous data were expressed as mean value ± SE, and compared between control and AAA groups using Student's t-test. Statistical significance of differences was defined as a P-value of <0.05. All statistical analyses were performed using SPSS 17.0 for Windows (SPSS Inc., IL, USA).

## Results

### Patient characteristics

Twenty AAA cases (16 male, 4 female; age 68.7±1.5 y) and 20 autopsy specimens (11 male, 9 female; age 66.5±3.0 y) were included in this study (Table 1). Male sex was more common among AAA patients compared with controls, but this difference was not statistically significant. Neither age nor BMI significantly differed between the AAA and control groups. The diameter of the abdominal aorta was larger in the AAA group than in the control group. The prevalence of hypertension, hypercholesterolemia, and cigarette smoking were more common in AAA group compared with the control group, but these differences were not statistically significant. The prevalence of hypertriglyceridemia, and diabetes mellitus were not statistically different between the AAA and control groups.

**Table 1:** Baseline characteristics of the AAA group and control group.

Category	Aneurysm (n = 20)	Control (n = 20)	*P* value
Sex (M/F)	16/4	11/9	0.176
Age	68.7±1.5	66.5±3.0	0.528
BMI	21.8±0.7	21.2±0.7	0.619
Diameter of abdominal aorta	49.8±2.7	18.0±0.7	<0.001
Hypertension (+/−)	15 (75%)/5 (25%)	11 (55%)/9 (45%)	0.320
Hypercholesterolemia (+/−)	9 (45%)/11 (55%)	5 (25%)/15 (75%)	0.320
Hypertriglyceridemia (+/−)	7 (35%)/13 (65%)	8 (40%)/12 (60%)	1.000
Diabetes (+/−)	3 (15%)/17 (85%)	4 (20%)/16 (80%)	1.000
Smoking (+/−)	15 (75%)/5 (25%)	11 (55%)/9 (45%)	0.320

The definition of each item: hypertension (medication for hypertension or a systolic blood pressure ≧140 mmHg and/or a diastolic blood pressure ≧90 mmHg), hypercholesterolemia (medication for hypercholesterolemia or total cholesterol concentration in the serum of ≧220 mg/dL), hypertriglyceridemia (medication for hypertriglyceridemia or total triglyceride in the serum of ≧150 mg/dL), diabetes (present or past medication for diabetes), smoking (present or past smoking history).

### Lymphatic microvessels in the intima of abdominal aortic aneurysm

**Figure 1:**
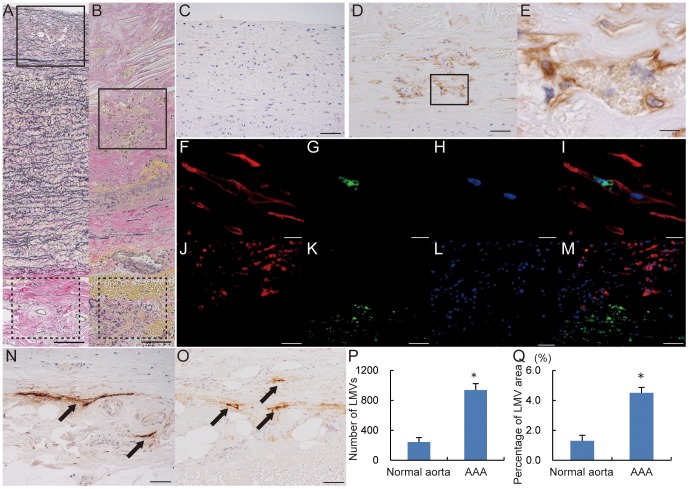
Lymphatic microvessels in intima of abdominal aortic aneurysm. EVG staining of normal aorta (**A**) and abdominal aortic aneurysm (AAA) wall (**B**). Immunohistochemistry for podoplanin corresponding to the areas outlined with a solid line in **A** and **B** (**C**, normal aorta; **D**, AAA). Podoplanin-positive microvessels were markedly increased in the intima in AAA wall. **E**, Higher magnification of the outlined area in **D**. Double immunofluorescence staining of lymphatic vessels in intima of AAA (**F**–**I**). **F**, Podoplanin (red); **G**, Prox-1 (green); **H**, DAPI (blue); **I**, merge of podoplanin, Prox-1, and DAPI. Double immunofluorescence staining of smooth muscle cells in intima of AAA (**J–M**). **J**, podoplanin (red); **K**, alpha smooth muscle isoform of actin (green), **L**, DAPI (blue); **M**, merge of podoplanin, alpha smooth muscle isoform of actin, and DAPI. Immunohistochemistry for podoplanin corresponding to the areas outlined with a dotted line in **A** and **B** (**N**; normal aorta, **O**; AAA). Lymphatic vasa vasorum was observed in adventitia in both normal aorta (**N**) and AAA (**O**) (black arrow: LVV). Scale bars indicate 100 µm (**A, B**), 50 µm (**C, D, J–M, N, O**), and 10 µm (**E–I**). Lymphatic microvessel density; the mean number of lymphatic microvessel (LMVs) in 10 microscopic fields at high magnification (×200) (**P**). Percentage of lymphatic microvessel area in 10 microscopic fields at high magnification (×200) (**Q**). Lymphatic microvessel density and percentage of lymphatic microvessel area were compared between normal aorta and AAA. (*P<0.001).

Elastica van Gieson (EVG) staining of AAA walls revealed intimal thickening and degradation of the wavy structure of elastin fibers in media compared with normal aorta (Fig. 1A, B). Immunohistochemical staining indicated that podoplanin was rarely expressed in the intima of normal aorta and frequently expressed in that of AAA (Fig. 1C, D). Additionally, some podoplanin-positive cells in the intima of AAA formed vessels (Fig. 1E).

Double immunofluorescence staining revealed that the nuclei of vessel-forming, podoplanin-positive cells in the intima of AAA expressed Prox-1 (Fig. 1F–I). Colocalization of podoplanin and the alpha smooth muscle isoform of actin was not detected in the intima of AAA (Fig. 1J–M).

Immunohistochemical staining indicated that podoplanin was expressed in the adventitia of normal aorta and AAA. Immunohistochemical staining of adventitial lymphatic vessels seemed indicate no morphological changes between the walls of normal aorta and AAA (Fig. 1N, O).

Lymphatic microvessel density in the intima was 243.0±59.3 (vessels/10 fields) in the control group and 938.4±85.7 (vessels/10 fields) in AAA group (*P*<0.001; Fig. 1P). The percentage of lymphatic microvessel area was 1.3±0.4% in the control group and 4.5±0.4% in the AAA group (*P*<0.001; Fig. 1Q).

### Lymphangiogenesis and angiogenesis in AAA

**Figure 2:**
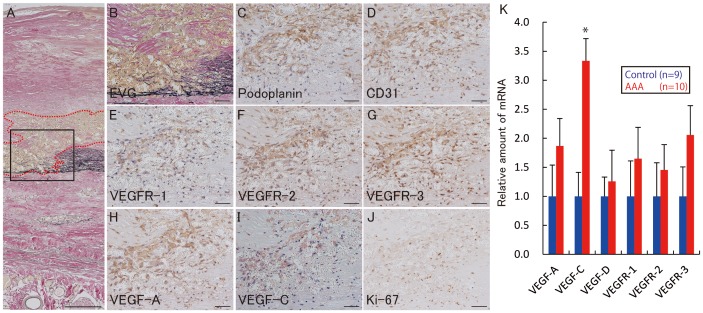
Lymphangiogenesis and angiogenesis in abdominal aortic aneurysm walls. **A**, Elastica van Gieson staining of the abdominal aortic aneurysm (AAA) wall. Microvessels in the intima/media are encircled by a red dotted-line. **B**, Higher magnification of the area outlined with a solid line in **A**. Immunohistochemistry for podoplanin (**C**), CD31 (**D**), VEGFR-1 (**E**), VEGFR-2 (**F**), VEGFR-3 (**G**), VEGF-A (**H**), VEGF-C (**I**), and Ki-67 (**J**) in the area outlined with a solid line in **A**. The microvessels encircled by the red dotted-line consisted of podoplanin-positive cells (**C**) and CD31-positive cells (**D**). These microvessels were positive for VEGFR-1, VEGFR-2, and VEGFR-3 (**E–G**). Expression of VEGF-A and C was increased within and around these microvessels (**H, I**). The nuclei of these microvessels were positive for Ki-67 (**H**). **K**, Comparison of mRNA expression of VEGF-A, VEGF-C, VEGF-D, VEGFR-1, VEGFR-2, and VEGFR-3 between normal aorta and AAA. Data were obtained from 10 AAA patients and 9 autopsied cases (controls). Data were analyzed by comparative Ct method. All of the mRNAs except VEGF-D were upregulated in AAA tissues. Standard deviation is indicated by bar errors. * indicates p<0.05 vs control. Scale bars indicated 200 µm (**A**), 50 µm (**B–J**).

EVG staining of AAA walls showed degradation of the wavy structure of elastin fibers in the media, where numerous microvessels were distributed from intima to media (Fig. 2A, B). Podoplanin-positive microvessels and CD31-positive microvessels were present (Fig. 2C, D). Furthermore, expressions of vascular endothelial growth factor receptor (VEGFR)-1, VEGFR-2, and VEGFR-3 increased in the microvessels (Fig. 2E–G). VEGF-A or VEGF-C- positive cells were also seen within and around the microvessels (Fig. 2H, I). The nuclei of microvessel cells demonstrated strong expression of Ki-67 (Fig. 2J). These microvessels were not seen in adventitia of AAA. Real-time quantitative PCR analysis indicated that mRNA expression of VEGF-A, VEGF-C, and VEGFR-1, VEGFR-2, and VEGFR-3 was upregulated in AAA tissue, while that of VEGF-D was not significantly different, in comparison with expression levels in normal aortic tissues (Fig. 2K).

### Infiltration of inflammatory cells into AAA walls

**Figure 3:**
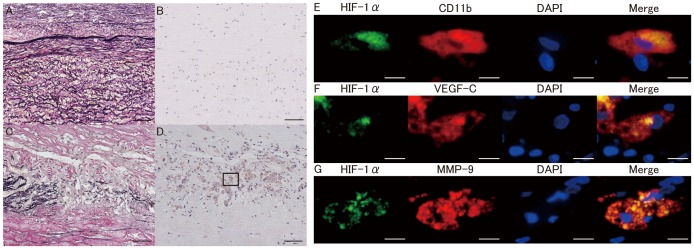
HIF-1 expression in normal aorta and AAA. Elastica van Gieson staining of the intima/media in normal aorta (**A**) and abdominal aortic aneurysm (AAA) wall (**C**). Immunohistochemistry for HIF-1α in normal aorta (**B**) and AAA (**D**). Nuclear and cytoplasmic expression of HIF-1α was observed in intima/media in AAA (**D**). **E**, Double immunofluorescence staining for HIF-1α (green), CD11b (red), DAPI (blue), and the merged image of the outlined in **D**. **F**, Double immunofluorescence staining for HIF-1α (green), VEGF-C(red), DAPI(blue), and the merged image of the outlined in **D**. **G**, Double immunofluorescence staining for HIF-1α(green), MMP-9 (red), DAPI (blue), and the merged image of the outlined in **D**. Expression of HIF-1α was increased in CD11b positive macrophages. VEGF-C and MMP-9 were expressed in the HIF-1α–positive macrophages. Scale bars indicated 50 µm (**A–D**) and 10 µm (**E–G**).

Comparison between normal aorta and AAA walls revealed marked degradation of elastin fibers in the media (Fig. 3A, C). Immunohistochemical staining showed both nuclear and cytoplasmic expression of hypoxia-inducible factor (HIF)-1α in the intima/media of AAA walls (Fig. 3D), while HIF-1α expression was not detected in that of normal aorta (Fig. 3B). Double immunofluorescence staining demonstrated infiltration of CD11b-positive macrophages with strong nuclear/cytoplasmic expression of HIF-1α into the intima/media of AAA(Fig. 3E). Those macrophages with strong nuclear/cytoplasmic expression of HIF-1α also expressed VEGF-C and MMP-9 (Fig. 3F,G)

**Figure 4:**
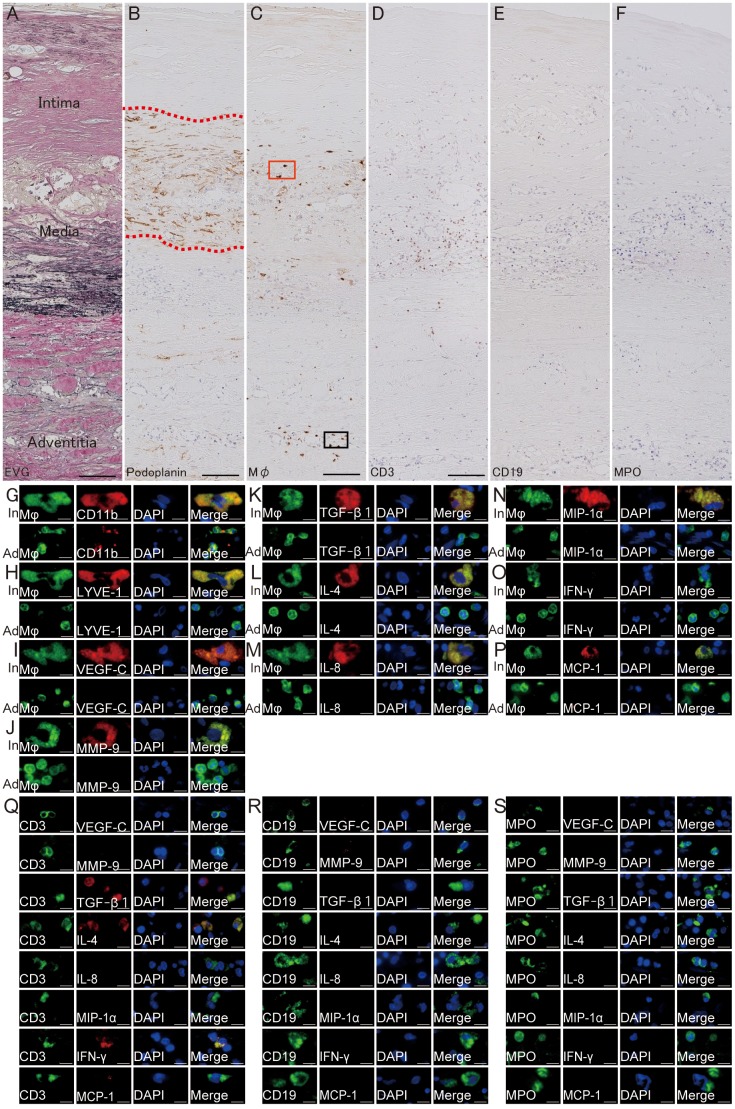
Macrophage infiltration of AAA microvessels. **A**, Elastica van Gieson staining of abdominal aortic aneurysm (AAA). Immunohistochemistry for podoplanin (**B**) and macrophages (**C**), CD19 (**E**), and myeloperoxidase (MPO) of the AAA wall. **B**, Lymphatic microvessels in the intima/media of the AAA (red dotted line encircling lymphatic microvessels). **C**, Macrophages infiltration around/within lymphatic microvessels in the intima/media or in adventitia (red square: macrophages in intima/media, black square: macrophages in adventitia). **D**, CD3-positive T cells infiltration around/within lymphatic microvessels in the intima/media or in adventitia. **E**, CD19-positive B cells infiltration around/within lymphatic microvessels in the intima/media or in adventitia. **F**, MPO-positive neutrophils infiltration in the intima/media or in adventitia. **G–P**, Double immunofluorescence staining of macrophages infiltrating the intima/media (In) and adventitia (Ad). **G–P**, Macrophage: green, CD11b (**G**)/LYVE-1 (**H**)/VEGF-C (**I**)/MMP-9 (**J**)/TGF-β1 (**K**)/IL-4 (**L**)/IL-8 (**M**)/MIP-1α (**N**)/IFN-γ (**O**)/MCP-1 (**P**): red, DAPI: blue. LYVE-1, VEGF-C, MMP-9, TGF-β1, IL-4, IL-8, MIP-1α, and MCP-1 were expressed in the CD11b-positive macrophages in the intima/media, but not by macrophages in adventitia. **Q**, Double immunofluorescence staining of T-cells in intima/media and inflammatory cytokines. CD3: green, VEGF-C/MMP-9/TGF-β1/IL-4/IL-8/MIP-1α/IFN-γ/MCP-1: red, DAPI: blue. TGF-β1, IL-4, and IFN-γ were expressed in CD3-positive T lymphocytes in the intima/media. **R**, Double immunofluorescence staining of B lymphocytes in the intima/media and inflammatory cytokines. CD19: green, VEGF-C/MMP-9/TGF-β1/IL-4/IL-8/MIP-1α/IFN-γ/MCP-1: red, DAPI: blue. These inflammatory cytokines were not expressed in CD19-positive B lymphocytes. **S**, Double immunofluorescence staining of neutrophils in intima/media and inflammatory cytokines. MPO: green, VEGF-C/MMP-9/TGF-β1/IL-4/IL-8/MIP-1α/IFN-γ/MCP-1: red, DAPI: blue. These inflammatory cytokines were not expressed in MPO-positive neutrophils. Scale bars indicated 100 µm (**A–F**) and 10 µm (**G–S**).

Using EVG and immunohistochemical staining of AAA walls, lymphatic microvessels were observed in the intima/media with degradation of elastin fibers (Fig. 4A,B). Inflammatory cells, such as macrophages, CD3-positive T lymphocytes, CD19-positive B lymphocytes, and neutrophils infiltrated in the surrounding intima/media andadventitia (Fig. 4C–F). Infiltration of these inflammatory cells was more prominent in the surrounding intima/media microvessels. Large macrophages were observed in the surrounding intima/media microvessels, while small macrophages were observed in the adventitia of AAA (Fig. 4G–M). Double immunofluorescence staining indicated that both types of macrophages (large macrophages in intima/media and small macrophages in adventitia) were positive for CD11b (Fig. 4G). However, only the large macrophages observed in the intima/media were positive for LYVE-1 (Fig. 4H). Similarly, large macrophages in the intima/media but not small macrophages in adventitia expressed VEGF-C, MMP-9, TGF-β1, IL-4, IL-8, MIP-1α, and MCP-1 (Fig. 4I, J, K, L, M, N, P). IFN-γ was not expressed either large macrophages in the intima/media or small macrophages in adventitia (Fig. 4P). CD3-positive T lymphocytes in the intima/media did not express either VEGF-C, MMP-9, IL-8, or MIP-1α, but expressed TGF-β1, IL-4, IFN-γ(Fig. 4Q).CD19-positive B lymphocytes and MPO-positive neutrophils in the intima/media did not express either VEGF-C, MMP-9, TGF-β1, IL-4, IL-8, MIP-1α, IFN-γ, and MCP-1(Fig. 4R,S).

### Lymph stasis in AAA walls

**Figure 5:**
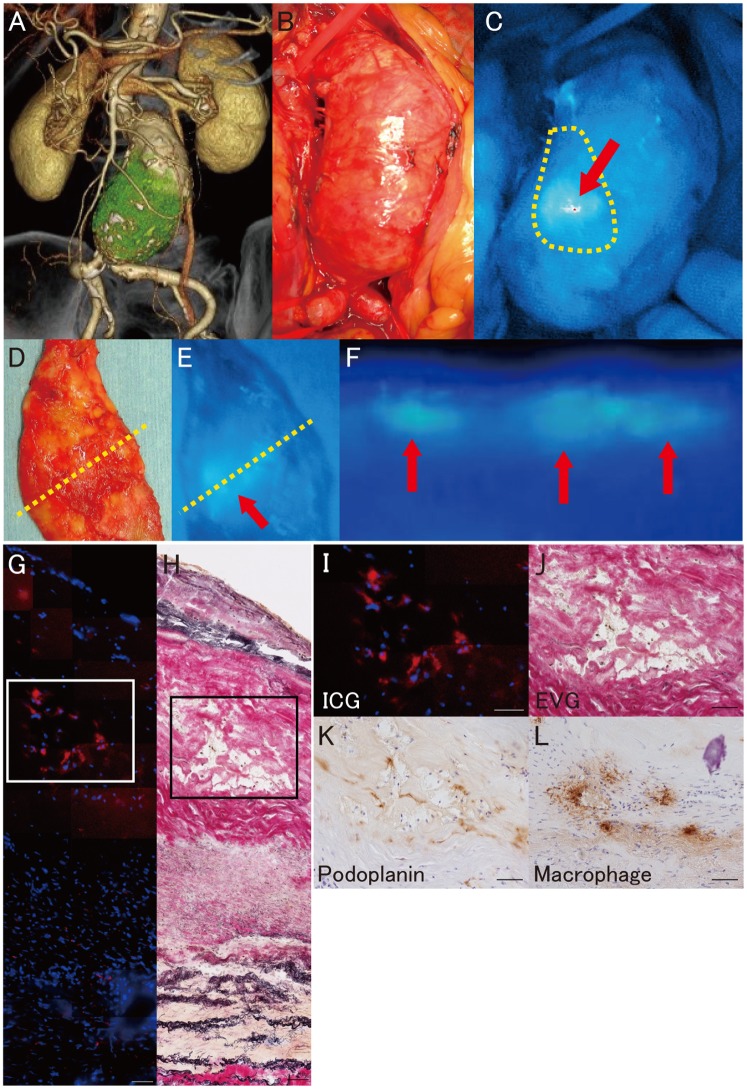
Fluorescence lymphography and microscopy of abdominal aortic aneurysm. **A**, Three dimensional CT image of an infrarenal abdominal aortic aneurysm (AAA). **B**, Intraoperative photography of an infrarenal AAA. **C**, Intraoperative near-infrared fluorescence lymphography of indocyanine green (ICG) (red arrow: ICG fluorescence, yellow dotted-line: sample resection line). **D**, Macroscopic findings of the resected sample observed from the luminal side (yellow dotted line: cross-section line of the harvested sample). **E**, Near-infrared fluorescence lymphography of ICG in the resected aneurysmal wall observed from the luminal side (red arrow: ICG fluorescence, yellow dotted line: cross-section line of the harvested sample). **F**, Near-infrared fluorescence lymphography of ICG in the cross-sectioned AAA wall (red arrow: ICG fluorescence, up: luminal side, bottom: adventitial side). **G**, Fluorescence microscopy of ICG in the AAA wall (ICG: red, DAPI: blue). **H**, Serial frozen section with Elastica van Gieson staining. **I**, Higher magnification of the outlined area in **G**. **J**, Higher magnification of the outlined area in **H**. **K**, Immunohistochemistry for podoplanin in the outlined area in **G** and **H**. **L**, Immunohistochemistry to label macrophages in the outlined area in **G** and **H**. Scale bars indicate 50 µm (**G–L**).

During open surgical repair of infra-renal AAA, ICG fluorescence lymphography was observed intraoperatively with a near-infrared camera after exposure of the retroperitoneal space to investigate lymph stasis, and strong ICG fluorescence signals were observed in the aneurysmal wall (Fig. 5 A–C). The pattern of the fluorescence-signal distribution was heterogeneous. ICG fluorescence lymphography was performed in all 17 patients with AAA. ICG fluorescence signals were identified in the AAA wall of all patients. Fluorescence lymphography of cross-sections of the aneurysmal wall in resected samples macroscopically localized the fluorescence signals to the intima/media area of the AAA wall (Fig. 5D–F). Near-infrared fluorescence microscopy also localized ICG fluorescence in the intima/media but not in adventitia, suggesting that lymph stasis occurred in the intima/media (Fig. 5G, H). Immunohistochemical staining revealed both extensive infiltration of macrophages and the presence of lymphatic microvessels in the intima/media of the AAA wall with lymph stasis (Fig. 5I–L).

## Discussion

Lymph transportation network in aortic wall tissue is largely unknown. Recently, Drozdz et al. first reported the presence of adventitial LVV in human aorta with an immunohistological technique [19]. In the present study, we also demonstrated the presence of adventitial LVV in both autopsied normal aorta and AAA. There seemed to be no morphological differences in adventitial LVV in AAA. The LVV is an innate vascular structure for lymph transportation, although the detailed role of LVV in aortic tissue is yet to be identified. Immunohistological examination also revealed intimal/medial lymphangiogenesis in the AAA wall, particularly in the area where medial elastin was marketly degraded. While lymphatic vessels are usually absent from normal aortic intima and media, podoplanin-positive microvessels significantly increased in AAA intima/media. Podoplanin is specifically expressed by lymphatic endothelial cells but not blood vascular endothelial cells [20]. Podoplanin has also been reported in normal cells other than lymphatic endothelial cells, such as peritoneal mesothelial cells, osteocytes, glandular myoepithelial cells, ependymal cells, stromal reticular cells and follicular dendritic cells [21], and was observed in intimal smooth muscle cells in atherosclerotic aorta [22]. In the present study, podoplanin expression was seen in the intima/media of AAA, but not co-localized with smooth muscle cells. The nuclei of podoplanin-positive cells that formed vessels expressed Prox-1. Therefore, we speculated that the podoplanin-positive microvessels were lymphatic microvessels arising from lymphangiogenesis.

Lymphangiogenic factors such as VEGF-C and VEGFR-3 were also present in the areas in which podoplanin-positive microvessels were observed. CD11b-positive macrophages were observed within and around lymphatic microvessels in both intima and adventitia of AAA walls. However, LYVE-1-positive macrophages were only observed in intima, not in adventitia. Previous studies have reported that macrophages are related to lymphangiogenesis in pathological processes such as malignant tumors [23], inflammation [24]–[26], and transplantation [27]. Macrophages are thought to support lymphangiogenesis in two ways: by transdifferentiation into lymphatic endothelial cells [23], [25]–[27], and by stimulating preexisting local lymphatic endothelial cells via release of lymphangiogenic factors [24], [25].Although LYVE-1 is regarded to be specifically expressed by lymphatic endothelial cells [20], LYVE-1-positive macrophages have also been reported in inflammatory tissue and malignant tumors [23], [26], [27]. Maruyama et al. recently reported that CD11b positive macrophages expressed LYVE-1 under inflammatory conditions in the cornea of mice. These macrophages aggregated and formed vessel-like structures, that expressed lymphatic endothelial cell markers, such as LYVE-1, Prox-1, and podoplanin [26]. CD11b and LYVE-1- positive macrophages may transdifferentiate into lymphatic endothelial cells. These macrophages could be derived from the circulation and transmigrate through the connective tissues, contributing to de novo lymphangiogenesis [25]–[27].

In the present study, overexpression of HIF-1α was observed in the intima/media of AAA, and large macrophages infiltrated hypoxic areas in the region. As we mentioned previously, the infrarenal aortic wall is susceptible to hypoxia. The presence of intraluminal thrombus (ILT) may prevent luminal perfusion of oxygen, allowing only the adventitial VV to deliver oxygen and nutrients to the aortic wall. Moreover, we recently reported that adventitial VV becomes stenotic due to the intimal hyperplasia, which causes malperfusion of the AAA wall and tissue hypoxia [5]. Notably, the tissue in the intima/media of AAA with thick ILT is farthest from both luminal perfusion and VV perfusion, so those layers are particularly prone to hypoxia. Vorp et al. also reported that thicker ILT was associated with localized tissue hypoxia, neovascularization, and inflammation in the AAA wall [28]. We therefore speculate that LYVE-1-positive macrophages may be induced by hypoxia in aortic intima/media and may transdifferentiate into lymphatic endothelial cells following lymphangiogenesis.

Macrophages can also promote lymphangiogenesis by producing lymphangiogenic factors, thereby stimulating the preexistent lymphatic endothelial cells in inflammatory tissue or in malignant tumors [23], [24]. The development of lymphatic vessels is regulated either by lymphangiogenesis-specific or angiogenesis-nonspecific factors in malignant tumors [24], [29]. Macrophage infiltration stimulates lymphatic endothelial cells by releasing VEGF-C. VEGF-C/VEGFR-3 signaling plays a critical role in the growth and survival of lymphatic endothelial cells in inflammation or in malignant tumors [25], [29]. In the present study, lymphatic microvessels were observed in the intima/media of AAA walls, where mRNA expressions of both VEGF-C and VEGFR-3 were more prominent. Expression of VEGF-C was also increased in HIF-1α positive macrophages infiltrating the intima/media but not in adventitial macrophages. Furthermore, the expression of VEGFR-3 was increased in areas of neovascularization in the intima/media of AAA walls. Taken together, these results suggested that hypoxia-induced infiltration of macrophages in intima/media may release VEGF-C, stimulating the lymphatic endothelial cells and thus contributing to lymphangiogenesis.

In this study, VEGF-A positive cells were also observed within and around the microvessels in both intima and media of AAA walls. The mRNA expression of VEGF-A and VEGFR-1 was more prominent than that in normal aorta. Kaneko et al. also reported VEGF-A overexpression in the macrophages infiltrating AAA walls [30]. Because VEGF-A is not only a potent angiogenic factor but also promotes lymphangiogenesis [24], [29], the VEGF-A/VEGFR-1 signaling pathway may also play an important role in both angiogenesis and lymphangiogenesis in the AAA wall.

Infiltration of inflammatory cells such as macrophages, T and B lymphocytes, and neutrophils, have been identified in AAA specimens [31]. In atherosclerotic lesions, monocyte-derived macrophages and T lymphocytes were observed most often [32], [33]. During each phase of atherogenesis, the inflammatory response is mediated by monocyte-derived macrophages and specific subtypes of T lymphocytes [34]. With regard to macrophages, Stoger et al. reported that, atherosclerotic lesions contain foamy large macrophages expressing M1 markers in the intimal plaque, while adventitial small macrophages express M2 signature karkers [35]. Similarly, in AAA specimens, both macrophages and several subtypes of T lymphocytes also have been identified [31]. In this study, the infiltration of large macrophages and T lymphocytes was confirmed in the intima/media, while small macrophages and T lymphocytes were observed in the adventitia of AAA. Only large macrophages in the intima/media of AAA expressed VEGF-C and MMP-9. Large macrophages may be associated with hypoxia in the intima/media, contributing to lymphangiogenesis and the progression of AAA. On the other hand, T lymphocytes and adventitial small macrophages did not express either VEGF-C or MMP-9, suggesting that T lymphocytes might not play a major role in involved in lymphangiogenesis in AAA walls. Infiltration of T and B lymphocytes and neutrophils was markedly observed around lymphatic microvessels, suggesting that formation of lymphatic microvessels may introduce these inflammatory cells into the intima/media. Macrophages in the intima/media expressed not only VEGF-C and MMP-9 but also TGF-β1, IL-4, IL-8, and MIP-1α. These cytokines are known to cause further recruitment of macrophages and neutrophils, potentiating inflammation [31], [36]. On the other hand, T lymphocytes expressed TGF-β1, IL-4, and IFN-γ but not VEGF-C and MMP-9, which suggested that T lymphocytes might accelerate macrophage recruitment in the region, but not directly contribute to the lymphangiogenesis [37], [38]. Inflammatory cytokines such as TGF-β1, IL-4, IL-8, and IFN-γ were reported to promote lymphangiogenesis in various diseases [39] [40] [41] [42]. Therefore, cytokines such as TGF-β1, IL-4, IL-8, and IFN-γ may also contribute to lymphangiogenesis in AAA. With regard to B lymphocytes in the intima/media, neither VEGF-C, MMP-9, TGF-β1, IL-4, IL-8, MIP-1α, IFN-γ, nor MCP-1 were expressed, which suggested that B cells may not play a major role in lymphangiogensis in AAA walls. Neutrophils did not express either VEGF-C and MMP-9, suggesting that neutrophils may contribute to macrophage recruitment rather than lymphangiogensis itself.

We investigated lymph stasis in AAA patients using ICG fluorescence lymphography. ICG fluorescence lymphography is an emerging technique to enable intraoperative observation of lymphatic flow [43], [44]. In the present study, ICG fluorescence dye, which was subcutaneously injected bilaterally into the dorsum of the foot, stagnated in the AAA wall. Intraoperative ICG lymphography using a near-infrared camera visualized marked fluorescence signals in the AAA wall after exposure of the retroperitoneal space. Freshly harvested samples of the cross-sectioned AAA walls also demonstrated fluorescence signals in the intima/media, which was later confirmed using ICG fluorescence microscopy. These results suggest that lymph stasis occurred in the intima/media of AAA wall. This suggested that neovascularized lymph vessels were a less efficient lymph-transport pathway, causing lymph stasis in the intima/media of AAA wall. In the harvested samples, the lymphedematous regions in the AAA wall also demonstrated marked infiltration of macrophages. Previous studies have associated chronic lymph stasis with tissue inflammation [45]. Recently, Zampell et al. reported that lymph stasis causes infiltration of mononuclear cells and tissue fibrosis in a lymphatic fluid stasis model using rat tails [46], [47]. Therefore, AAA wall lymphatic dysfunction causes lymph fluid stasis, which may contribute to the medial inflammation in the AAA wall [47]. Hypoxia-induced lymphangiogenesis could also be enhanced by lymph stasis [48]. However, the role of lymphangiogenesis in the AAA wall is unknown. Angiogenesis is thought to contribute to destructive processes within the AAA wall and plays a key role in aortic aneurysm development and rupture [7]. Therefore, various anti-angiogenic agents have been studied in animal models for potential treatment of AAA [30], [49], [50]. Maruyama et al. reported that down-regulation of VEGFR-3 and subsequent inhibition of lymphangiogenesis delayed diabetic wound healing in a mouse model [51]. Moreover, Zhou et al. reported that administration of VEGF-C and increased lymphangiogenesis improved lymph drainage and inhibited inflammation in a rat model of chronic arthritis [52]. Thus, inflammation has been widely accepted to stimulates lymphangiogenesis as a compensatory mechanism to enhance the clearance of inflammatory products. Therefore, lymphanagiogenesis in the AAA wall may be a rational response against loss of adventitial LVV and lymph stasis. However, as we demonstrated in the present study using ICG fluorescence lymphography, the newly formed lymphatic vessels may not fully function to drain lymph. Then, the insufficient lymph drainage may provoke further inflammation.

In conclusion, macrophage infiltration may be associated with lymphangiogenesis and angiogenesis in the intima/media. Lymph-drainage appeared to be insufficient in the AAA wall, which may become a new therapeutic target for non-surgical treatment of AAA by improving lymph flow.
